# Resilience to Disturbance Despite Limited Dispersal and Self-Recruitment in Tropical Barrel Sponges: Implications for Conservation and Management

**DOI:** 10.1371/journal.pone.0091635

**Published:** 2014-03-20

**Authors:** James J. Bell, David Smith, Danielle Hannan, Abdul Haris, Jamaludin Jompa, Luke Thomas

**Affiliations:** 1 School of Biological Sciences, Victoria University of Wellington, Wellington, New Zealand; 2 Coral Reef Research Unit, University of Essex, Colchester, United Kingdom; 3 Research and Development Center on Marine, Coastal and Small Islands, Hasanuddin University, Makassar, Indonesia; 4 The Oceans Institute, School of Plant Biology, University of Western Australia, Australia; University of Genova, Italy, Italy

## Abstract

While estimates of connectivity are important for effective management, few such estimates are available for reef invertebrates other than for corals. Barrel sponges are one of the largest and most conspicuous members of the coral reef fauna across the Indo-Pacific and given their large size, longevity and ability to process large volumes of water, they have a major role in reef functioning. Here we used a panel of microsatellite markers to characterise the genetic structure of two barrel sponge species, *Xestospongia testudinaria* and a currently undescribed *Xestospongia* species. We sampled across seven populations in the Wakatobi Marine National Park, SE Sulawesi (Indonesia) spanning a spatial scale of approximately 2 to 70 km, and present the first estimates of demographic connectivity for coral reef sponges. Genetic analyses showed high levels of genetic differentiation between all populations for both species, but contrasting patterns of genetic structuring for the two species. Autocorrelation analyses showed the likely dispersal distances of both species to be in the order of 60 and 140 m for *Xestopongia* sp. and *Xestospongia testudinaria*, respectively, which was supported by assignment tests that showed high levels of self-recruitment (>80%). We also found consistently high inbreeding coefficients across all populations for both species. Our study highlights the potential susceptibility of barrel sponges to environmental perturbations because they are generally long-lived, slow growing, have small population sizes and are likely to be reliant on self-recruitment. Surprisingly, despite these features we actually found the highest abundance of both barrel sponge species (although they were generally smaller) at a site that has been severely impacted by humans over the last fifty years. This suggests that barrel sponges exhibit environmental adaptation to declining environmental quality and has important implications for the management and conservation of these important reef species.

## Introduction

Coral reefs across the world have been seriously impacted by human activities to the extent that many reefs have been severely degraded [1]. Furthermore, local-scale impacts such as increases in sedimentation, habitat destruction and overfishing, coupled with global scale threats such as global warming and ocean acidification provide an uncertain future for coral reefs ecosystems [2]. However, while there has been considerable focus on how corals and fish respond to such degradation and might be impacted by future climate change scenarios, far less is known about other important reefs organisms, and little information is available to support their conservation and management. Connectivity is widely recognised as a key component of reef resilience and population viability [3]–[4]. Connectivity is a broad term that can be further split in to: 1) genetic connectivity, which refers to the degree to which gene flow affects evolutionary processes within populations; and 2) demographic connectivity, which refers to the degree to which population growth and vital rates are affected by dispersal and recruitment [5]. A key step in measuring demographic connectivity is to determine the actual numbers of individuals that are exchanged between populations, but this is very difficult for marine species as most rely on a pelagic larval phase to link populations. Understanding patterns of exchange between populations is important because its controls a population’s buffering potential from local catastrophes, a population’s potential as a source of new individuals to other populations and the level of genetic mixing between populations [6]–[7]. Generally, environmental managers are most interested in demographic connectivity as it controls processes over the temporal scales that management typically operates (years to decades).

Genetic techniques have been widely applied to estimate patterns of genetic connectivity, and more recently demographic connectivity of tropical marine species, but the focus has primarily been on corals and fish [8]–[11], with little information on connectivity patterns of other reef organisms. In order to understand broader patterns of connectivity for biologically complex reef systems it is important to understand the exchange patterns of other major components of tropical reef systems. Without such information it will not be possible to effectively manage entire reef systems.

Sponges are common and important coral reef organisms across the world with a range of important roles in ecosystem functioning [12], key roles include the ability of sponges to process vast quantities of water consuming picoplankton, Dissolved Organic Carbon and potentially viruses, bioerosion of carbonate substrate, and supporting rich macro and microbial communities [12]–[14]. Furthermore, recent research suggests that sponges may be one of the few taxonomic groups to benefit from the long-term trends declines coral reefs [15]–[17]. Little is known about the spatial scales at which sponge populations are interconnected across reef systems; this represents a major gap in our knowledge of the functional ecology and dynamics of both healthy and degraded systems, and the role sponges play in these systems. In fact there are comparatively few estimates of genetic connectivity for sponges [18]–[20] and no estimates of demographic connectivity for coral reef sponges (but see [21] for a sub-tropical species).

Of the 10,000+ species of known sponges, one of the most conspicuous and charismatic are the giant barrel sponges of the genus *Xestospongia*, which can reach several meters in diameter and have been suggested to reach several thousand years in age [22]. Given their size and common occurrence, they are likely to be one of the more important sponge species on Indo-Pacific reefs [12]. While *Xestospongia muta* from the Caribbean has been one of the most intensively studied sponge species (e.g., [21]–[24]), far less is known about the *Xestospongia* species from the Indo-Pacific (but see [25]–[27] despite barrel sponges being commonly found across the region. Currently, two barrel sponge species have been described from the Indo-Pacific, (*X. testudinaria* and *X. bergquistia*), which have been separated on the basis of morphology [25] and sterol chemistry [26]. While the reproductive biology has been extensively studied in *X. testudinaria* and *X. bergquistia*, with both species being gonochoristic and oviparous, the length of the larval period is unknown, but is likely to be short (hours to days) based on studies of other sponge species [28]. Until recently *X. testudinaria* has been considered a single species, but a recent study by Swierts et al. [29] found evidence for a species complex within what has been considered *X. testudinaria*. With a combination of mitochondrial and nuclear markers and a morphological analysis, these authors proposed that there are at least two distinct *Xestospongia* species around Lembeh Island, Indonesia, each with different habitat preferences.

Given the slow growth and small census population sizes of barrel sponges, they would appear susceptible to environmental degradation and disturbance. However, large, old individuals are common on reefs suggesting that other species traits allow populations to persist. This may be explained by their ability to exist across a range of environmental conditions, their role as strong spatial competitors (through upward rather than highly competitive horizontal growth), their potential to tolerate environmental disturbances, or as a result of high levels of population connectivity. Identifying these traits will be increasingly important as predicted future reef impacts provides more opportunities for benthic species other than coral to dominate reef systems. We recently described the development of a panel of microsatellite markers for *Xestospongia* spp. [30], with the initial results from these markers providing evidence to support the division of barrel sponges into genetically distinct groups and likely species. Based on this earlier analysis and Swierts et al. [29], here we explore data across a great geographic range within an Indonesian archipelago and characterise the genetic structure of two putative *Xestospongia* species. We aimed to: 1) measure and compare the levels of genetic and demographic connectivity between populations of the two species of *Xestospongia* at a range of spatial scales (2 km to 70 km); 2) estimate dispersal distances using an spatial autocorrelation analysis and assignment testing; 3) examine differences in the level of genetic diversity and inbreeding at sites with different environmental conditions; and 4) compare the abundance and size distributions at sites with different environmental and biological characteristics to consider the impacts of disturbance on population structure. The life-history characteristics and population demography of barrel sponges would appear to make sensitive to disturbance. Therefore we hypothesise that barrel sponges will show low levels of dispersal, high levels of self-recruitment and therefore must have evolved physiological traits enabling them to tolerate degraded and disturbed environments. Understanding levels of connectivity will provide insights into how these functionally important reef organisms should be managed and conserved.

## Materials and Methods

### Sample Sites

**Figure 1:**
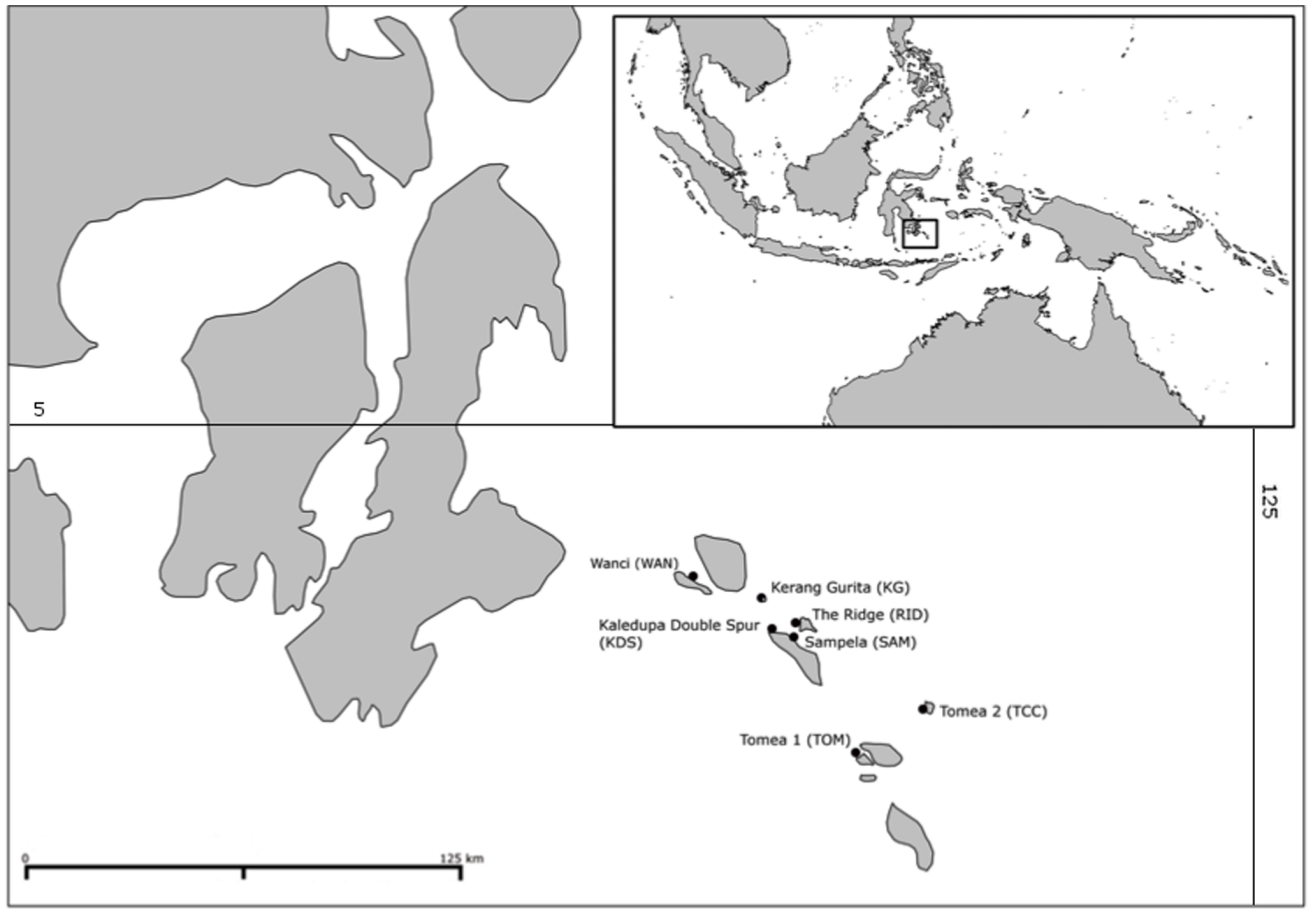
Sampling locations of *Xestospongia* spp. in the Wakatobi Marine National Park.

Seven sites (see Fig. 1) were sampled in the Wakatobi Marine National Park (WMNP) in southeast Sulawesi in Indonesia in June 2012 under permit number 0212/SIP/FRP/SM/V11/2011 from the Ministry of Research and Technology (RISTEK). The WMNP was gazetted in 1996 and is the third largest marine national park in Indonesia [31]. The park is located in the coral triangle and supports some of the world’s most diverse marine communities, but is also inhabited by over 90,000 people who are heavily reliant on reef resources [32]. There is a large variation in the coral cover, fish abundance and environmental conditions at sites across the park, and many areas have suffered major declines in coral cover over the last 10 years [33]. Sites were classified based on observations of live coral cover, water clarity, distance to major human populations, fish abundance, evidence of bomb fishing and degree of coral bleaching/disease. Based on our observations (at 6–18 m depth), the sites in Wanci Harbour and at Samplea were considered to be the lowest quality sites based on being close to a large population (<1 km), experiencing heavy sedimentation and having very low coral cover (5–10%) and fish abundance. The sites at Kaladupa Double Spur, the Ridge and Tomea 1, were considered to have moderate site quality with good water clarity, moderate coral cover (25–35%) and some distance from large human populations (3–5 km), while the sites at Karang Gurita and Tomea 2 where considered to be the highest quality sites with high water clarity, high coral cover (>35%) and the longest distance (5–10 km) to the nearest large population. Observations and site categorisation are consistent with the findings of the long term monitoring programme within the study area [33].

All barrel sponges were sampled in an area approximately 250 m long between 6 and 21 m at each site, except Sampela, where the maximum depth of the reef extends to 12 m, giving a total sampling area (and population size for the site) of 2400 m^2^ at each site except Sampela where 1200 m^2^ was surveyed. Sampling was depth restricted in line with safe diving practises (<21 m) but some barrel sponges were observed below this depth threshold. One cm^3^ tissue samples were collected from each sponge and preserved in 99% ethanol. Photographs and measurements were also taken of each sponge.

The restricted reef formation reef at Sampela (depth restricted to 12 m) allowed us to sample the entire population along a 250 m section of the reef. This survey was used for a spatial autocorrelation analysis (see below), where we mapped the location of each barrel sponge on the reef relative to each other sponge by running a transect along the base of the reef at 12 m and determining x,y coordinates (in metres) along the reef for each sponge.

### DNA Extraction and Microsatellite Amplification

Genomic DNA was extracted from the ethanol preserved *Xestospongia* tissue samples using an Isolate Genomic DNA Mini Kit (Bioline) following the manufacturer’s protocols. We used the nine of the twelve markers developed Bell et al. [31] for *Xestospongia* (three markers were discarded based on initial screening that detected null alleles at two loci and evidence of selection acting on one locus).

Microsatellite loci were amplified for each sponge sample on a GeneAmp 2700 (Life Technologies) thermocycler in a final reaction volume of 12 μl containing: ∼50 ng template DNA, 1X Bioline MyTaq Red Mix (0.11 units/μl TaqDNA polymerase, 82.5 mM Tris–HCl pH 8.5, 22 mM (NH_4_)_2_SO_4_, 1.65 mM MgCl_2_, 0.22 mM dNTPs), equal amounts of forward and reverse primer (see 31), and ddH_2_0 to volume. The following cycle conditions were used: 94°C for 5 minutes; followed by 40 cycles of 94°C for 30 seconds, 60°C for 45 seconds, 72°C for 60 seconds; followed by a final extension at 72°C for 10 minutes. PCR products were visualised on a 1.5% agarose gel using ethidium bromide staining and if successfully amplified were genotyped on an ABI3730XL (Life Technologies) automated capillary sequencer. The 5′ ends of the forward primers were tagged with the fluorescent labels FAM, VIC, NED or PET. Multiplex Manager 1.2 [34] was used to arrange loci into two multiplex PCR panels.

### Preliminary Analyses

Because de Swierts et al. [19] have proposed a species complex for *Xestospongia testudinaria*, we first conducted an exploratory analysis of our entire dataset to determine whether or not there was support for dividing our data, representing two or more species. In addition, we genotyped archived specimens from the Queensland Museum (Australia) representing *X. testudinaria* (G320630, G322086, G322647, G321612, G321769, G324859, G25009, G25010, G25011, G25012) and *X. bergquistia* (G25018, G25019) from the Indo-Pacific. To visualise the genetic relationships between all samples from all populations, a Principle Coordinate Analysis (PCA) was conducted by genealex 6.3 using the pairwise matrices of Nei’s genetic distance [35]. Based on the results of this plot, and subsequent examination of the morphologies of each sponge sampled (from photographs), we conducted all analyses separately for the two genetically distinct groups previously identified by Bell et al. [31]. To support this, we used a hierarchal analysis of molecular variance (AMOVA, n = 10 000 permutations) to determine the proportion of genetic variation that could be attributed to differences between the two main groups identified from the PCA using genealex v 6.3.

### Genetic Diversity

Allele discovery curves were generated using PopGenKit Package in the software R 2.13.1 for all Samples from Sampela. Curves that reach an asymptote indicate that the allelic diversity would not be increased by analysing additional samples. We quantified genetic diversity within each of the sampled populations for each species based on the mean number of alleles per locus and total number of alleles using fstat v 2.9.3 [36] and Nei’s unbiased heterozygosity [37] with genealex 6.3 [38]. We also preformed exact tests to identify any deviations from Hardy Weinberg equilibrium with genealex v 6.3 and tested for any evidence of linkage disequilibrium between all loci/population combinations with arlequin v 3.5.1.2 [39] P-values were adjusted for multiple comparisons using standard Bonferroni corrections [40]. Markov-chain parameters were 10 000 dememorization steps, 1 000 batches and 10 000 iterations per batch.

### Population Genetic Structure

We used a hierarchal analysis of molecular variance (AMOVA, n = 10 000 permutations) to determine the proportion of genetic variation that could be attributed to differences between sampling sites using genealex v 6.3. Population differentiation (*F_ST,_* [41]) was also measured across all loci with arlequin v 3.5.1.2 using sampling locations as population units. Significance values were based on 10 000 permutations. There is much debate as to whether the Stepwise Mutation Model (SMM) and associated *R_ST_* index is appropriate for population genetic studies using microsatellite loci [42]; therefore, we avoided using the *R_ST_* index. A Mantel test for Isolation by distance was conducted using linearized *F_ST_* (*F_ST_*/(*1−F_ST_*) and minimum oceanographic distance (km) between sites.

To visualise the genetic relationships between sampled populations, a Principle Coordinate Analysis (PCA) was conducted by genealex 6.3 using the pairwise matrices of *F_ST_* and Nei’s genetic distance (D_s_).

Bayesian clustering analysis in structure v 2.3.2 [43] was used to infer population structure using the admixture model with correlated allele frequencies among clusters and informed priors with a burn-in period of 10^4^ iterations and 10^5^ Markov Chain Monte Carlo (MCMC) repetitions and with K ranging from 1–7. Each individual in the data set is represented by a single vertical line, which is partitioned into K segments that represent that individual’s estimated membership fraction in each of the K inferred clusters. The appropriate K value for the data set was determined by plotting the log probability (L(K)) and ΔK across multiple runs [44] as implemented in structure harvester [45]. Results from 10 runs were merged with clumpp [46] and visualized using distruct [47].

Assignment tests were conducted using a Bayesian approach [48] in geneclass v 2.0 [49]–[50] and an analysis of first generation migrants was conducted using an exclusion threshold approach where individuals were excluded from their corresponding sampling site when probability of assignment to the reference population was less than 0.05 (Type I error; 50). This exclusion approach is likely to be more accurate than the “leave one out” methodology by Paetkau et al. [51] because it does not require that all true source populations be sampled, and in our case it is likely there are un-sampled populations. Excluded individuals were then reassigned to a source population when the probability of assignment was greater than 10%. When an excluded individual was re-assigned to more than one population (*P*>0.10) it was left unassigned. Those individuals that could not be re-assigned to any of the other populations were considered to have originated from a non-sampled location.

### Spatial Autocorrelation Analysis

To investigate fine-scale patterns of genetic relatedness, spatial autocorrelation tests were performed by GENEALEX 6.4.1 [52] to determine if the degree of genetic similarity between individual sponges was correlated with the geographic distance between them. This analysis was conducted using pairwise matrices of genetic distance and geographic distance across all loci among sponges from Sampela, where the specific location of each individual sponge was recorded along a 250 m transect (see above). The spatial autocorrelation coefficient of genetic distance (r) was calculated over 15 distance classes (up to 150 m) and illustrates the genetic similarity of individuals whose pairwise geographic distance falls within a specific distance class. The location where r crosses the x-intercept provides an estimate of positive spatial genetic structure [52], where genetic drift rather than gene flow is the primary force influencing spatial structure. For each distance class 95% confidence intervals were generated based on 10000 permutations and 10000 bootstrap replicates.

### Abundance and Size of Sponges

As we sampled all sponge specimens along a 250 m section of reef at each location, this provided us with an estimate of total population size at each location. At Karang Gurita and Sampela (considered to be one of the highest and lowest environmental quality sites, respectively) we also measured the approximate size of each barrel sponge. We generalised the shape of each sponge to a cylinder with a cone cut from the top to represent the spongocoel. We measured the maximum circumference, the height of the sponge, the diameter of the spongocoel and depth of the spongeocoel to calculate the approximate volume.

## Results

### Preliminary Genetic Analyses

**Figure 2:**
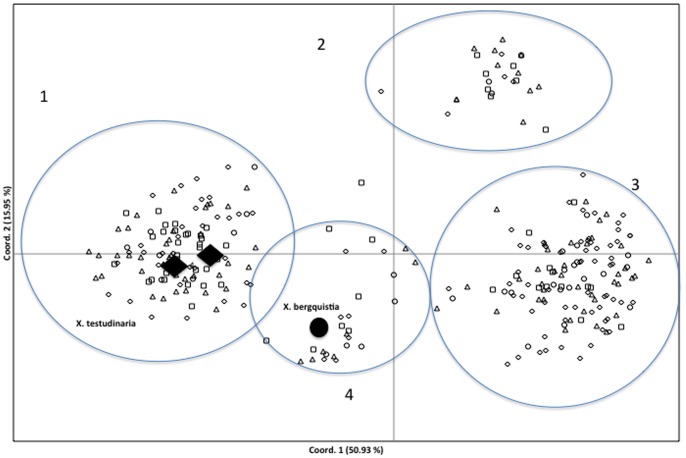
Principle Coordinate Analysis (PCA) implemented in GENEALEX v 6.3 of pairwise (A) Nei’s genetic distance for all samples from the Wakatobi. The museum specimens of *Xestospongia testudinara* and *X. bergquistia* are shown as large symbols.

The PCA to examine the relationships between all of the samples across all seven sites showed clusters of samples, which were not consistent with their geographical location, and formed four distinct groups (Figure 2). AMOVA showed that 50% of the variation (P<0.010) could be attributed to among group differences. On re-examination of the photographs of each sampled sponge, those that fell within three of the groups could generally be distinguished based on their external morphology and we propose these likely represent different species (consistent with [19]). For the four group it was difficult to distinguish individuals based on morphology. There were no apparent differences between the geographical locations of the samples in any of the groups and all groups appeared to have the same distribution patterns. Only two of the groups had sufficient sample sizes to enable further analyses and the subsequent analyses were conducted separately for these two species (i.e. the sponges from the two other clusters were not included in any further analyses). Inclusion of the museum specimens within the PCA enabled us to determine whether any of the groups were consistent with the two described barrel sponges species, *Xestospongia testudinaria* and *X. bergquistia* (Figure 2). Unfortunately (presumably due to DNA degradation), it was only possible to amplify the microsatellites for two of the *X. testudinaria* samples and one of the *X. bergquistia* samples. The two *X. testudinaria* samples clustered with one of the two larger groups supporting that these samples are *X. testudinaria*, while the single *X. bergquistia* sample clustered with one of the smaller groups, suggesting the second large cluster from the Wakatobi may represent an undescribed species (hereafter referred to a *Xestospongia* sp.).

### Genetic Diversity

**Figure 3:**
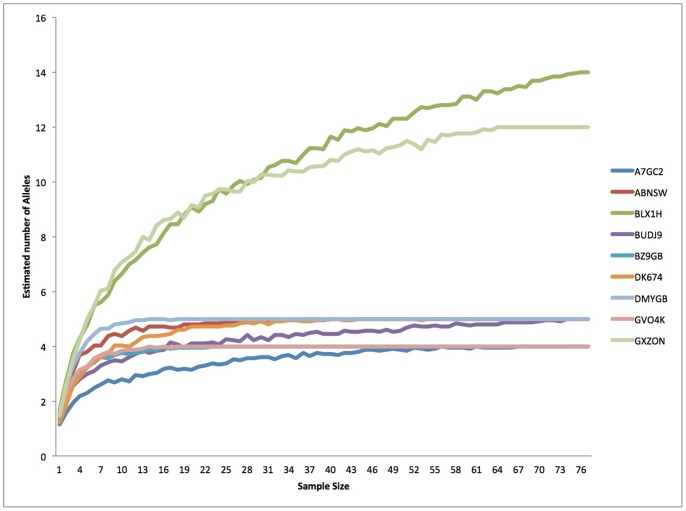
Allele discovery curves for each locus using samples from the Sampela site, which as the largest population size.

Allele discovery curves (Figure 3) showed that for most loci an asymptote was reached at low sample sizes (<10). We found no evidence of linkage disequilibrium between any pair of loci across all sample sites following standard Bonferroni corrections for either species. All populations exhibited a significant global heterozygote deficiency (Table 1) with inbreeding coefficients (*F_IS_*) ranging from 0.131 to 0.431 for *X. testudinaria* and 0.054 to 0.364 for *Xestospongia* sp. We found deviations from HWE at the locus level varied between sampling sites, although no population showed a significant deficiency at all loci (Table 2). The mean number of alleles per locus ranged from 3 to 4 for *X. testudinaria* and 2.77 to 4.33 for *Xestospongia* sp., while the mean number of alleles per population ranged from 3 to 4 for both species (Table 2). Locus A7GC2 was monomorphic in *Xestospongia* sp. Genetic diversity was lowest at Tomea (TOM) for *X. testudinaria* and Karang Gurita (KG) for *Xestospongia* sp. (Table 2). Comparisons of each genotype with each other genotype across all our samples revealed no evidence of clones within the sampled populations (i.e. no two sponges sampled had the same genotype).

**Table 1:** Sampling locations within the Wakatobi Marine National Park with standard genetic diversity indices.

a) Group 1							
Population	Coordinates	N	N_A_	H_O_	H_E_	F_IS_	*P*_HWE_
Sampela (SA)	36° 34′ S 174° 46′ E	28	4.00	0.47	0.57	0.131	0.001
Wanci (WAN)	46° 38′ S 167° 37′ E	10	3.00	0.42	0.53	0.186	0.030
Karang Gurita (KG)	43° 55′ S 176° 43′ W	21	3.78	0.45	0.56	0.192	0.001
Kaledupa Double Spur (KDS)	41° 20′ S 174° 48′ E	8	3.00	0.31	0.54	0.431	0.001
Ridge (RID)	42° 24′ S 173° 40′ E	5	3.89	0.51	0.67	0.248	0.080
Tomea 1 (TCC)	43° 53′ S 166° 09′ E	19	3.89	0.43	0.58	0.229	0.001
Tomea 2 (TOM)	36° 49′ S 139° 49′ E	17	3.56	0.35	0.48	0.271	0.001
**b) Group 2**		**N**	**N_A_**	**H_O_**	**H_E_**	**F_IS_**	***P*** **_HWE_**
Sampela (SA)	36° 34′ S 174° 46′ E	46	4.11	0.375	0.49	0.212	0.001
Wanci (WAN)	46° 38′ S 167° 37′ E	7	3.44	0.515	0.54	0.054	0.116
Karang Gurita (KG)	43° 55′ S 176° 43′ W	20	3.33	0.369	0.45	0.186	0.001
Kaledupa Double Spur (KDS)	41° 20′ S 174° 48′ E	19	4.33	0.449	0.53	0.167	0.001
Ridge (RID)	42° 24′ S 173° 40′ E	14	4.33	0.498	0.51	0.067	0.06
Tomea 1 (TCC)	43° 53′ S 166° 09′ E	5	2.77	0.289	0.46	0.364	0.006
Tomea 2 (TOM)	36° 49′ S 139° 49′ E	17	3.55	0.398	0.49	0.149	0.002

(N) number of individuals sampled; (N_A_) mean number of alleles per locus; (H_O_) observed and (H_E_) expected heterozygosity; and (F_IS_) inbreeding coefficients with corresponding significant values (P_HWE_).

**Table 2:** Allele size variation (standardized allelic richness) and total number of alleles per locus (N) at nine microsatellite loci.

Group A	SA	WAN	KG	KDS	RID	TCC	TOM
**locus A7GC2**	4	3	4	3	4	4	4
**locus ABNSW**	2	2	3	3	4	4	4
**locus BLX1H**	10	6	7	5	6	6	4
**locus BUDJ9**	3	3	4	3	4	5	4
**locus BZ9GB**	3	3	2	2	3	2	2
**locus DK674**	3	2	4	3	3	3	3
**locus DMYGB**	3	3	3	2	5	4	6
**locus GVO4K**	3	3	3	2	2	2	2
**locus GXZON**	5	2	4	4	4	5	3
Mean	4	3	4	3	4	4	4
Total	36	27	34	27	35	35	32
UHe	0.578	0.561	0.571	0.580	0.747	0.592	0.497
**Group B**	**SA**	**WAN**	**KG**	**KDS**	**RID**	**TCC**	**TOM**
**locus A7GC2**	1	1	1	1	1	1	1
**locus ABNSW**	3	4	3	3	6	3	4
**locus BLX1H**	8	6	5	6	5	5	4
**locus BUDJ9**	4	3	4	5	5	2	2
**locus BZ9GB**	4	3	4	5	4	3	5
**locus DK674**	4	2	2	3	2	2	3
**locus DMYGB**	4	3	3	5	4	3	3
**locus GVO4K**	2	2	2	3	4	2	2
**locus GXZON**	7	7	6	8	8	4	8
Mean	4	3	3	4	4	3	4
Total	37	31	30	39	39	25	32
UHe	0.496	0.584	0.461	0.542	0.540	0.509	0.501

Genetic diversity indices including total number of alleles per population, mean number of alleles per loci, and Nei’s unbiased Heterozygosity (UHe), are also shown.

### Population Genetic Structure

**Figure 4:**
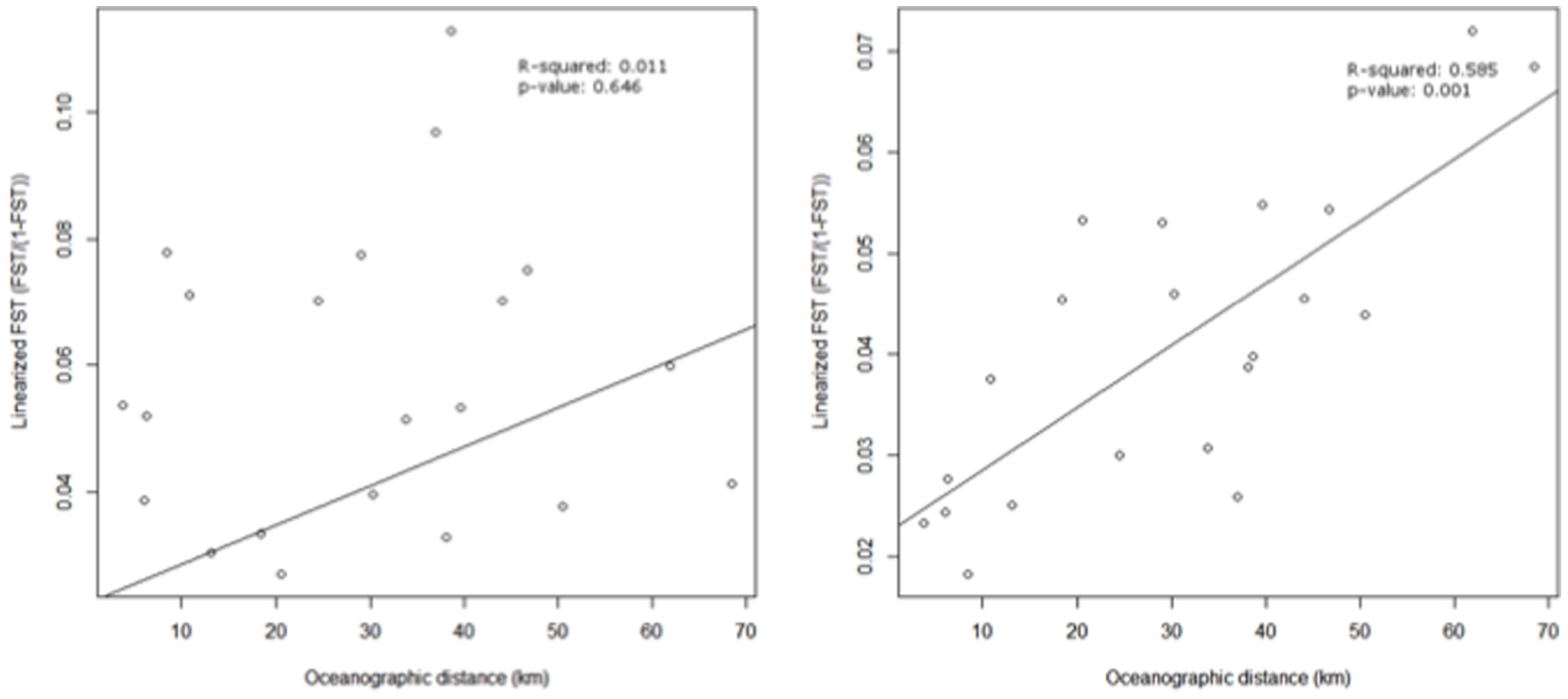
Mantel test of Isolation by distance using linearized *F_ST_* with 95% confidence intervals for the two groups (Group – left, Group B – right).

Significant *F_ST_* values were detected for all pairwise comparisons following standard Bonferroni corrections (Table 3). Isolation by distance plots (Figure 4) showed no relationship for *X. testudinaria* (R^2^ = 0.01, P = 0.65), but a strong significant positive correlation for *Xestospongia* sp. (R^2^ = 0.68, P<0.001). Our analysis revealed significant population structure for both species across the Wakatobi (Global *F_ST_* of 0.054, P = 0.010 for *X. testudinaria*; Global *F_ST_* of 0.039, P = 0.010 for *Xestospongia* sp.). Irrespective of species, AMOVA indicated only a small proportion (7 and 5% for *X. testudinaria* and *Xestospongia* sp., respectively; Table 4) of genetic variation could be attributed to differences between sampling sites.

**Table 3:** Pairwise fixation index values F_ST_ for group A (above diagonal) and group B (below diagonal).

	SA	WAN	KG	KDS	RID	TCC	TOM
Sampela (SA)		0.038	0.029	0.037	0.051	0.032	0.049
Wanci (WAN)	0.044		0.032	0.065	0.072	0.040	0.056
Karang Gurita	0.024	0.043		0.072	0.066	0.036	0.070
Kaledupa DS	0.024	0.029	0.018		0.049	0.066	0.101
Ridge (RID)	0.023	0.050	0.036	0.027		0.051	0.088
Tomea 1 (TCC)	0.037	0.064	0.042	0.044	0.052		0.026
Tomea 2 (TOM)	0.030	0.067	0.052	0.038	0.025	0.051	

Bold values indicate significance based on 10,000 permutations with a Bonferroni adjusted *P* value of 0.001.

**Table 4:** Hierarchal analysis of molecular variance (AMOVA) was used to estimate levels of genetic differentiation between sampling sites (n = 7) for the two groups (P<0.01).

	Source	df	SS	MS	Est. Var.	%
Group A	Among Populations	6	92.240	15.373	0.543	7%
	Within Populations	105	738.894	7.037	7.037	93%
	Total	111	831.134		7.580	100%
Group B	Among Populations	6	63.647	10.608	0.280	5%
	Within Populations	123	714.976	5.813	5.813	95%
	Total	129	778.623		6.093	100%

**Figure 5:**
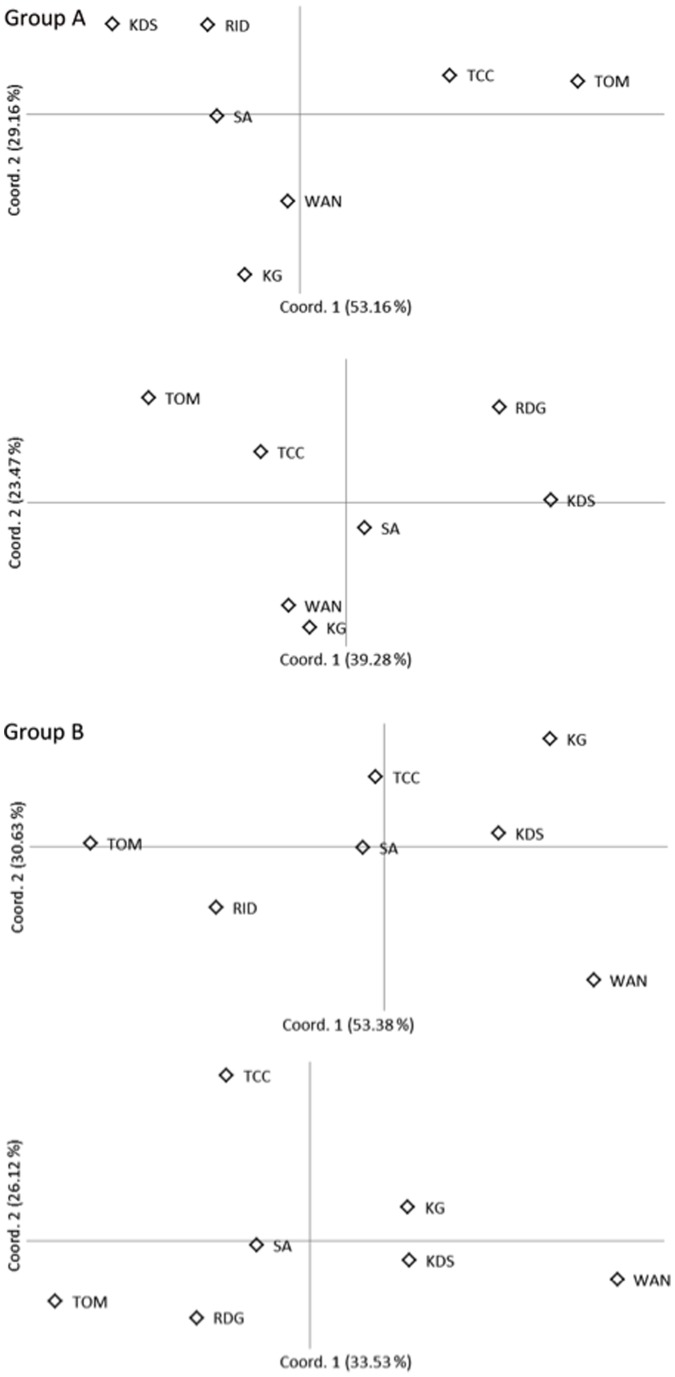
Principle Coordinate Analysis (PCA) implemented in GENEALEX v 6.3 of pairwise (Nei’s genetic distance (above) and *F_ST_* values (below) between populations of two groups (A and B) of *Xestospongia* samples collected in the Wakatobi Marine National Park.

PCA plots (Figure 5) show the genetic relationships between the sampled locations, and results were relatively consistent across the measures of differentiation (*D_s_* and *F_ST_*). For *X. testudinaria*, the PCA showed evidence of four main clusters including the sites to the south (TOM and TCC), the north (WAN and KG), a central group (RID and KDS) and Sampela. For *Xestospongia* sp., there were no obvious population clusters consistent with isolation by distance structuring.

**Figure 6:**
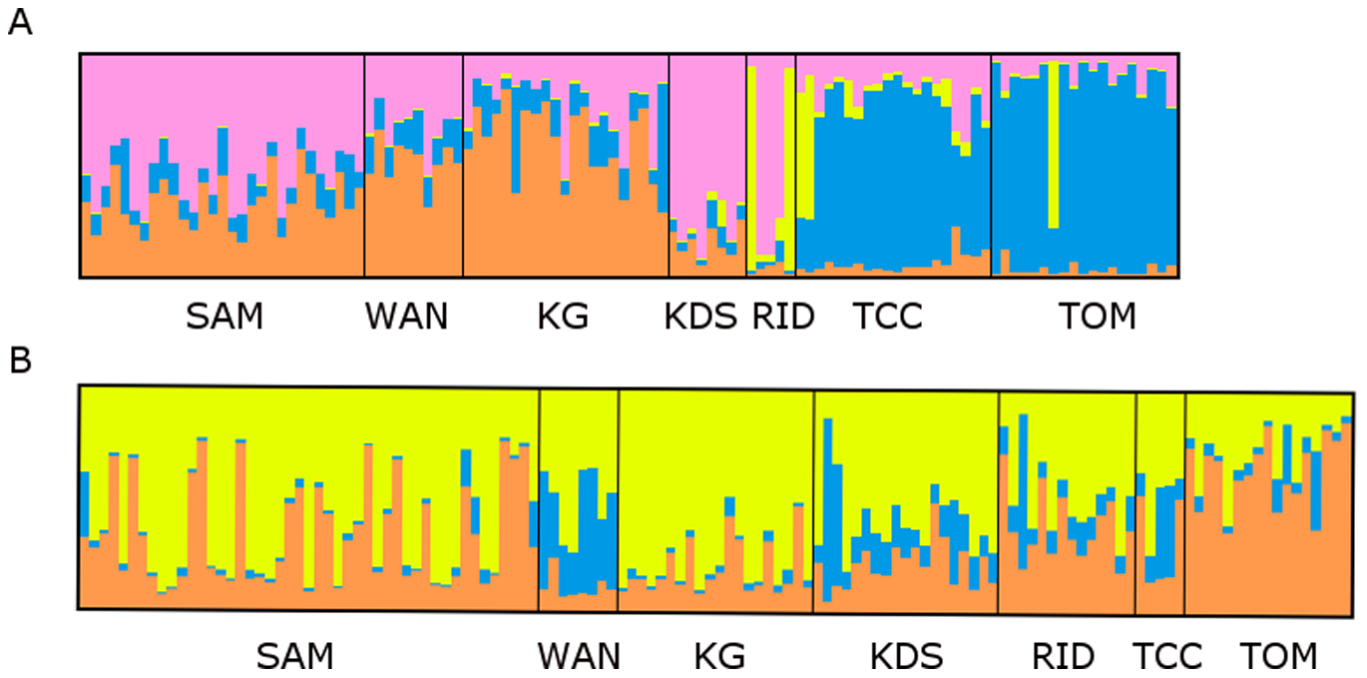
Bayesian clustering analysis using STRUCTURE 2.3.2 for two groups of *Xestospongia* samples (group 1– upper chart, A: group 2 lower chart, B). Sampling locations are along the x-axis and membership coefficient in each predefined cluster (K) is along the y-axis. K = 3 and K = 4 clustering scenarios according to STRUCTURE HARVESTER for group 1 and 2, respectively. Results were averaged across 10 runs with CLUMP and visualized with DISTRUCT.

**Figure 7:**
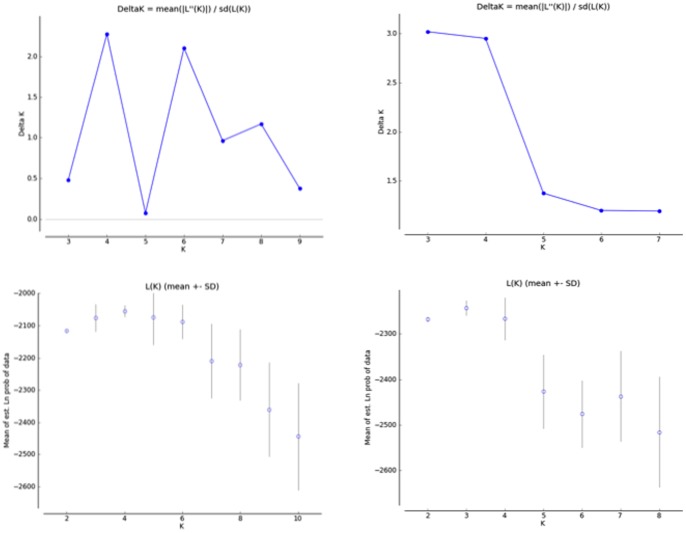
Plots generated in STRUCTURE Harvester that show the mean log likelihood of the data [L(K)] and Evanno’s delta K statistic for both sponge groups.

Differentiation patterns identified from PCA plots were supported by STRUCTURE. The optimal number of clusters according to Evanno et al. (2005) as implemented in STRUCTURE HARVESTER was determined to be K = 4 (Figure 6 and 7) for *X. testudinaria*: two central groups (SAM = 1; RID and KDS = 2), a northern group (KG and WAN) and a southern group (TOM and TCC). STRUCTURE HARVESTER identified K = 3 for *Xestospongia* sp., (SAM, RID and KDS = 2), a northern group (KG and WAN) and a southern group (TOM and TCC). However, this was not strongly supported (see Figure 6), which is consistent with a gradient and the isolation by distance patterns described above for this species.

### Assignment Testing and Self-recruitment

Our assignment testing identified high levels of self-recruitment for both species, although care is needed in the interpretation of the percentage of self-recruiting sponges for populations with small sample sizes (Table 5). For *X. testudinaria*, populations with sample sizes >10 generally had a low number of excluded individuals (<12%) indicating high levels of self-recruitment. There was also evidence of sponges that could not be assigned to any of our sampled populations, with only 2–3% of sponges being assigned to an unsampled population.

**Table 5:** Assignment tests conducted in GeneClass v. 2.0.

Population	# of Excluded Individuals	% of pop	Assigned Population							Not Assigned
			SAM	WAN	KG	KDS	RDG	TCC	TOM	
SAM (28)	1	3%	–	0	0	0	1	0	0	0
WAN (10)	1	10%		–		0	1	0	0	0
KG (12)	1	5%	0		–	0	1	0	0	0
KDS (8)	3	38%	0	0		–	3	0	0	0
RDG (5)	2	40%	0	0	0		–	0	0	2
TCC (19)	2	10%	0	0	0	0	2	–	0	0
TOM (17)	2	11%	0	0	0	0	2	0	–	0
Average		17% (+/−15)								
**Population**	**# of Excluded Individuals**	**% of pop**	**Assigned Population**							**Not Assigned**
			**SAM**	**WAN**	**KG**	**KDS**	**RDG**	**TCC**	**TOM**	
SAM (46)	4	9%	–	1	0	2	0	0	0	1
WAN (7)	1	13%	0	–	0	1		0	0	0
KG (20)	3	15%	0	1	–	0	2		0	0
KDS (19)	2	11%	0	0		–	0	0	0	2
RDG (14)	1	7%	0	0	0		–	0	0	1
TCC (5)	1	20%	0	0	0	0	1	–	0	0
TOM (17)	2	12%	0	0	0	1	0	0	–	1
Average		12%(+/−4)								

Detection of first generation migrants was done using an exclusion threshold approach where an individual was excluded from their corresponding sampling site when the probability of exclusion was >95%. Individuals that were excluded were assigned to another population when the probability of assignment was above a more conservative threshold of >0.10. Individuals that could not be re-assigned to any of the sampled populations with a p value >0.10 were considered to have originated from a non-sampled location. Numbers in brackets in column 1 are the total sample sizes for each population.

### Spatial Autocorrelation Analyses

**Figure 8:**
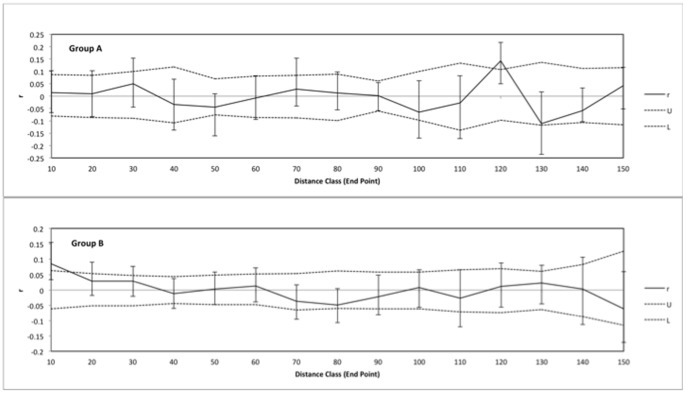
Spatial autocorrelation analyses of the genetic correlation coefficient (r) as a function of distance for two genetically distinct groups of *Xestopongia* on the Wakatobi Marine National Park calculated with GenAlEx v6 (Peakall and Smouse 2006). The bootstrapped 95% confidence error bars generated via 1000 bootstrap trials are shown.

Our spatial autocorrelation analysis revealed small differences in the likely dispersal distances between the two groups at Sampela (Figure 8). Results for *X. testudinaria* indicated that genotypes were distributed randomly for the first 5 distance classes (20–100 metres), after which the *r* value showed a significant positive correlation (*p = 0.035*) among genotypes for sponges separated by distances of 120 metres. The correlation coefficient then dropped to become significantly negative (*p = *0.011) at 140 metres. Spatial autocorrelation analysis of *Xestospongia* sp. indicated a significant positive correlation (*p* = 0.008) among genotypes of sponges within the first distance class (20 metres). Values then levelled of between the next two classes and dropped below the *x-*axis to become significantly negative (*p = *0.021) among genotypes of sponges separated by distances greater than 80 metres. Values were again significantly negative (*p = *0.004) at 160 metres. These results indicated that the neighbourhood size of this *X. testudinaria* was approximately 140 m and for *Xestospongia* sp. approximately 65 metres (based on the location where *r* crosses the *x-*axis; [53]).

### Abundance and Size of Sponges

**Figure 9:**
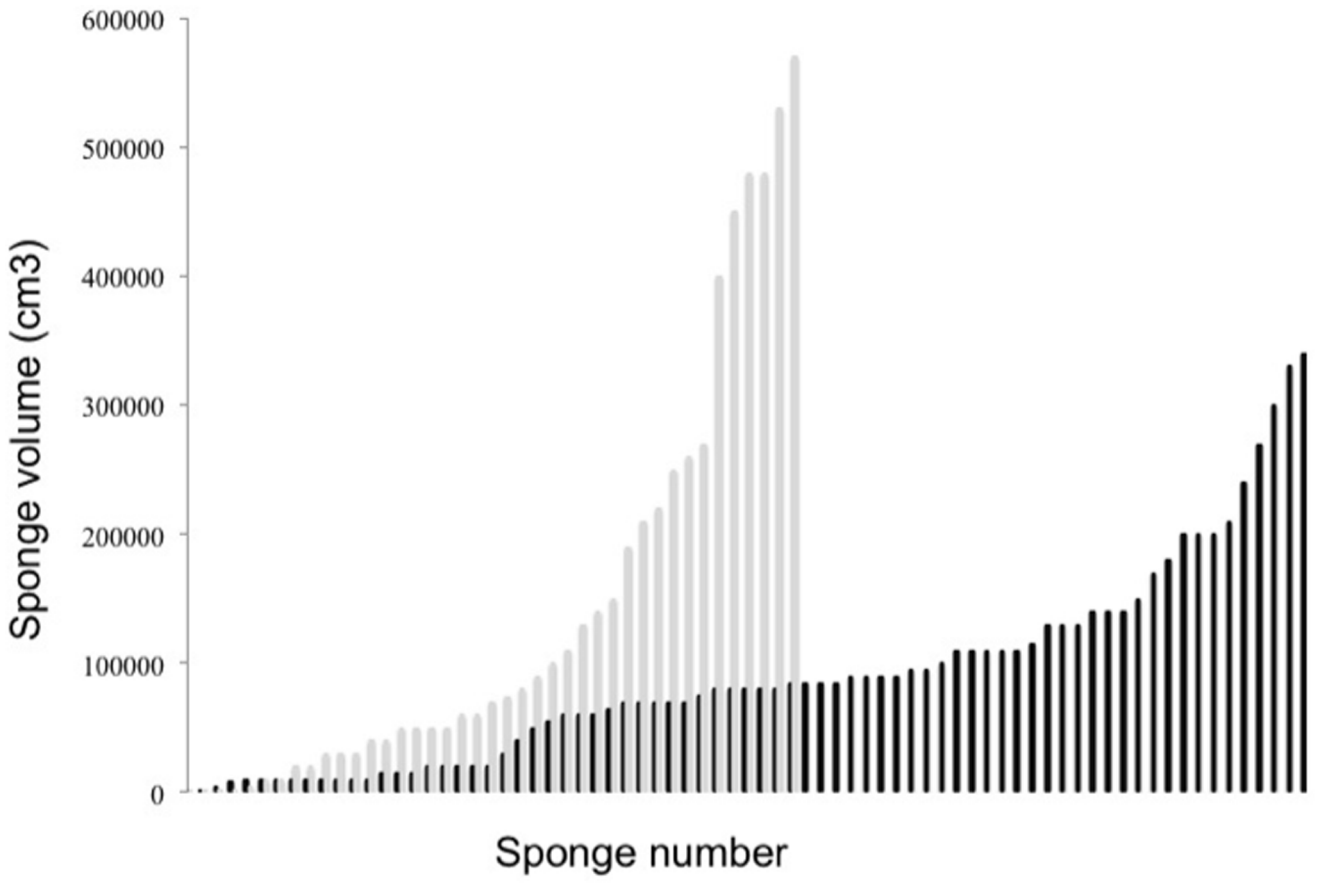
Size distribution of all barrel sponges (all specimens) along a 250 m section of reef at two sites in the Wakatobi Marine National Park. Grey lines are data from Karang Gurita (KG) and black lines for Sampela (SAM).

Estimates of population size for the two species were only based on the densities of a single section of each reef (which we consider sites), but based on this we found the overall (across both species) highest abundance of sponges at Sampela and the lowest abundance at the Ridge (Table 1). However, there appeared to be no general pattern between abundance and reef quality based on our qualitative site assessments. The size distribution of sponges showed that the sponges at Sampela were generally much smaller than those at Karang Gurita (Figure 9) and the overall sponge biomass (for a 1250 m^2^ section of reef) was lower at Sampela (337.60 m^3^ compared with 581.26 m^3^). At both these sites there appeared to be sponges of a range of size classes, suggesting recruitment has been relatively consistent. However, we did not find any small, very recent, recruits at ether site.

## Discussion

Despite connectivity being considered a key component in understanding population dynamics and in the development of effective management plans, we still have a very poor understanding of the processes that link populations and the scales at which populations are connected. This is especially the case for non-coral reef invertebrates. Here we distinguish between multiple co-occurring species of Indo-Pacific barrel sponges based on microsatellite data, which is supported by gross morphological differences with one of the species being *X. testudinaria*, another *X. bergquistia* and the other two are likely undescribed species. Our results support the presence of a recently proposed *X. testudinaria* species complex in the Indo-Pacific [19]. We aimed to measure the levels of genetic differentiation and demographic connectivity between populations for the two most abundant species. Our genetic data showed strong levels of genetic differentiation between populations separated by as little as 2 km for both species. Furthermore, the results of assignment tests and spatial autocorrelation analyses suggest barrel sponges generally have small dispersal distances of several hundred meters and are likely to be reliant on self-recruitment. However, assignment tests did find some sponges that could not be assigned to any sampled populations and sponges that had come from more distant populations suggesting some potential for occasional longer-distance dispersal. We found different types of genetic structuring for the two species, with *X. testudinaria* following an island model [54] and *Xestospongia* sp. an isolation by distance model [55]. High levels of self-recruitment coupled with their small population census sizes and long-lived nature would appear to make barrel sponges susceptible to disturbance. However, barrel sponges were most abundant at one of the most heavily disturbed reefs suggesting some adaptive ability to deal with environmental disturbance. These results support a recent hypothesis that some sponge species may become more abundant on coral reefs in response to climate change and ocean acidification [16], as they may also benefit from other types of environmental degradation that results in declines in potential sponge predators and competition.

### Demographic Connectivity

There are currently very few estimates of demographic connectivity for most reefs organisms other than corals and fish, and even for these groups estimates are rare. While larval duration has been proposed as a predictor of larval dispersal potential, there is increasing evidence that populations may also be dependent on a high level of self-recruitment. For example, using DNA parentage data and marking via stable isotopes, Almany et al. [56] found that approximately 30% of juvenile clownfish (*Amphiprion polymnus*) settled within 100 m of their natal reefs, despite having a relatively short (compared to other marine species) 12-day larval duration. Estimating demographic connectivity is difficult for most marine species because of the problems in tracking microscopic larvae through the oceans. Parentage, assignment testing and spatial autocorrelation analyses are powerful tools for estimating demographic connectivity, but they are best suited for species with small census population sizes and limited dispersal capacity [57]. These features of barrel sponges make them suitable for such analyses, and our findings suggests these species have short dispersal distances and high levels of self-recruitment. However, there are some problems with using our findings for barrel sponges to infer connectivity amongst other sponge species within the region. Generally, the majority of sponges in this region comprise small (<10 cm^2^) encrusting patches, whose densities can exceed hundreds of sponge patches per m^2^ [58]–[59] so have a much larger census population size their population demography will be very different. However, given sponges generally have short-lived pelagic larvae (in the order of hours to days; Maldonado 2006), dispersal distances are also likely to be small, and it seems likely they will also be reliant on self-recruitment. Interestingly, at the Sampela site, the sponge *Lamellodysidea herbacea* was the most abundant in 2003 and has almost doubled in abundance over the last decade [17], [58] (as coral has declined, 33). However, despite coral declines at other sites in the Wakatobi the same increases in *L. herbacea* have not been seen. Although environmental conditions at Sampela (heavy sedimentation and high turbidity) may favour this species, its rapid increase in abundance suggests a local stock-recruitment relationship, which is likely to be supported by self-recruitment.

### Interpretation of Genetic Patterns

We found strong patterns of genetic differentiation between all sites for both species. *X. testudinaria* followed an island model, with three different groups consistent with their geographic position, while *Xestosponiga* sp. followed an isolation by distance model. Currently nothing is known of the oceanographic features of the Wakatobi or the location of any potential barriers to gene flow, so it is difficult for us to explain these patterns. However, given we found different patterns of genetic structuring for the two co-occurring barrel sponge species suggests different process are influencing gene flow between populations of these species. *X. testudinaria* had a larger estimated dispersal distance than *Xestospongia* sp. (120 m compared with 65 m), although these distances are still small compared to species with long-lived pelagic larvae, which might be in the order of 10 s to 1000 s km [60]. It is therefore possible that other ecological (e.g. longevity and fecundity) or physiological factors account for the differences between species. However, earlier reproductive studies [27] found little difference in the reproductive biology between *X. testudinaria* and *X. bergquistia*, although larval duration and behaviours have not yet been studied extensively; this might account for the differences we reported. We found high inbreeding coefficients for nearly all populations that we sampled for both groups, with an apparent excess of homozygotes, but no patterns between sites. While null alleles might account for this result, this seems unlikely as MICROCHECKER found no evidence for null alleles in the loci that we used (see Bell et al. [30] for discarded loci showing evidence of null alleles and selection). Given the small census population size, limited dispersal distances and consistency of this pattern across sites, it seems likely that inbreeding might account for these heterozygote deficiencies. This is also supported by individual loci showing few deviations from HWE.

Our results are consistent with a number of earlier genetic studies on sponges using allozymes and microsatellite markers. For example, Whalan et al. [19] found significant genetic differentiation between populations of *Haliclona* sp. over different spatial scales using allozymes, including differences between populations only several hundred meters apart. Currently, microsatellite markers have only be applied to two other sponge species, the Mediterranean species’ *Crambe crambe* [18] and *Scopalina lophyropoda* [21]. Again these studies highlight high levels of genetic differentiation between populations separated at small (100 m) spatial scales. Furthermore, both these studies found high inbreeding coefficients within sponge populations and proposed asexual reproduction and inbreeding as possible explanations. While sponges are well known to reproduce through asexual propagation, we found no evidence for clones within our data and therefore propose inbreeding as the most likely cause of the high *F_IS_* values that we reported. A genetic analysis of the related barrel sponge *Xestospongia muta* in the Caribbean using the I3-M11 partition of the Cytochrome Oxidase (COI) gene found high levels of genetic differentiation between populations at spatial scales of several 100 km [20], nearly 1000 times the scale of our study. However, Given the conserved nature of the marker used by these authors they were unable to examine genetic differentiation at small-scale levels (100 m to kms). Interestingly, like our results for *X. testudinaria*, these authors also found no evidence of isolation by distance, and proposed that while limited larval dispersal may have led to differentiation among some of the populations, the patterns of genetic structure appear to be most strongly related to patterns of ocean currents.

One of the primary limitations to our study is the small sample size for many of our populations. The identification of multiple *Xestospongia* species was unexpected during the initial collection of specimens for this study, but meant we had to split our data into two groups. Furthermore, some sponges were excluded from the analysis (n = 65) as they appeared to represent different species. As a result some care is needed in the interpretation of our data. However, the relatively low numbers of alleles for each of our loci based on an initial survey of 75 individuals [30] means it is unlikely there is a large amount of undetected variation within the populations we sampled; this is also supported by our allele discovery curves (Figure 3). Furthermore, our evidence for strong genetic differentiation between sites is supported by the autocorrelation analysis based on large single site sample size showing short dispersal distances giving confidence to our results.

It is interesting that we found evidence for possibly four *Xestospongia* sp. species, and this was consistent with differences in the external morphology of these specimens. The taxonomic relationships between these species will be the focus of future studies. Our study is not the first to report possible undescribed species within a *Xestospongia testudinaria* complex [19]. It is possible that one of the *Xestospongia* species that was not included in the genetic analysis does have a different local habitat distribution, as specimens were mainly collected from deeper water (21 m).

### Management and Conservation Implications

Barrel sponges play important functional roles through their ability to filter large volumes of water [12]. Our results demonstrate limited dispersal, high levels of self-recruitment and strong levels of genetic differentiation between populations of two *Xestospongia* species. Their small population size coupled with these features should make barrel sponges susceptible to environmental disturbance or there must be a large selective pressure for barrel sponges to tolerate a range of environmental conditions and be able to tolerate environmental perturbations. Sampela has experienced major declines in coral cover and fish in recent years [33], yet this site was where we found the highest numerical abundance of barrel sponges (although lower overall biomass). However, the size distribution of those sponges indicated they were generally smaller at Sampela compared to those at Karang Gurita. Size has been considered an indicator of age, based on growth models created for the Caribbean barrel sponge *Xestospongia muta* [22]. Although there are likely to be local and regional differences in growth rates and these are as yet are unqualified, extrapolation of these growth models from the Caribbean to sponges in the Wakatobi makes the largest sponges at Sampela to be around 50–60 years old and 250–300 years old at Karang Gurita. Interestingly, these ages at Sampela correspond to the settlement of a Bajo village very close to the Sampela reef that was only settled in the 1950s, and has been heavily exploited ever since. It is unclear if these events are related or whether size differences between sites are a functional of differential growth rates at the different sites, but this warrants further investigation and demonstrates the potential resilience of barrel sponges to environmental degradation and potential ability to recover following disturbance.

The development of management plans and any assessment of system resilience requires an understanding of connectivity patterns. However, determining the probability of inter-population connectivity needs to incorporate population dynamics and the factors that control larval longevity and survival. Such information is essential for managers, but collecting this information is time consuming and is generally impractical for all reef species. However, genetic data can be used to infer patterns of genetic and demographic connectivity, but it needs to be interpreted in the context of the species’ ecology. Given that the degree of reef resilience can be altered through increased connectivity [3]–[4], without knowledge of dynamics, and population structure it is impossible to determine appropriate spatial scales of protection, conservation priority areas and likely outcomes of disturbance. Managers need to focus on enhancing system resilience either indirectly through the precautionary principle or directly through prioritising the protection of areas that are sources of potential recruits. Thus greater knowledge is required concerning the fundamental population biology of key reef organisms and in particular those species that play important functional roles.

In the case of barrel sponges, populations appear largely reliant on self-recruitment, which should have important implications for their management, which coupled with their long-lived nature and apparent low level of recruitment would appear to make them susceptible to physical disturbance or fatal disease outbreaks. Despite this, barrel sponges are abundant at sites considered to have experienced high levels of degradation in the past, suggesting some level of tolerance to disturbance. Furthermore, these sites also experience heavy levels of sedimentation, suggesting some level of adaptation to these conditions. While this might appear something of a paradox, the features of barrel sponge populations means they are likely to require management at relatively local-scales (several km) as population extinctions are unlikely to be replenished by distant larval sources.

### Conclusions

Here we report the first estimates of demographic connectivity in tropical sponges, and demonstrate barrel sponges in the Wakatobi have low dispersal distances and are likely to be heavily reliant on self-recruitment, with high levels of inbreeding. The features of these species’ would make them susceptible to disturbance but this does not appear to be the case. This provides further evidence to support the hypothesis that sponges may be likely ‘winners’ in response to any further environmental degradation.

## References

[1] Burke L, Reytar K, Spalding M, Perry A (2011) Reefs at Risk Revisited. World Resources Institute, Washington DC. 400 p.

[2] Hoegh-Guldberg O, Mumby PJ, Hooten AJ, Steneck RS, Greenfield P, et al (2007) Coral reefs under rapid climate change and ocean acidification. Science 318: 1737. 18079392 10.1126/science.1152509

[3] Hughes TP, Baird AH, Bellwood DR, Card M, Connolly SR, et al (2003) Climate change, human impacts, and the resilience of coral reefs. Science 301: 929–933. 12920289 10.1126/science.1085046

[4] Hughes TP, Graham NA, Jackson JB, Mumby PJ, Steneck RS (2010) Rising to the challenge of sustaining coral reef resilience. TREE 25(11): 633–642. 20800316 10.1016/j.tree.2010.07.011

[5] Lowe WH, Allendorf FW (2010) What can genetics tell us about population connectivity? Mol Ecol 19: 3038–3051. 20618697 10.1111/j.1365-294X.2010.04688.x

[6] Allison GW, Gaines SD, Lubchenco J, Possingham HP (2003) Ensuring persistence of marine reserves: Catastrophes require adopting an insurance factor. Ecol App 13: 8–24.

[7] Bell JJ, B Okamura (2005) Low genetic diversity in a marine nature reserve: re-evaluating diversity criteria in reserve design. Proc Royal Soc Biol Sci 272: 1067–1074. 10.1098/rspb.2005.3051 PMC1599875 16024366

[8] Jones GP, Almany GR, Russ GR, Sale PF, Steneck RS, et al (2009) Larval retention and connectivity among populations of corals and reef fishes: history, advances and challenges. Coral Reefs 28: 307–325.

[9] Saenz-Agudelo P, Jones GP, Thorrold SR, Planes S (2009) Estimating connectivity in marine populations: an empirical evaluation of assignment tests and parentage analysis under different gene flow scenarios. Mol Ecol 18: 1765–1776. 19243510 10.1111/j.1365-294X.2009.04109.x

[10] Underwood JN, Smith LD, Oppen MJV, Gilmour JP (2009) Ecologically relevant dispersal of corals on isolated reefs: implications for managing resilience. Ecol App 19: 18–29. 10.1890/07-1461.1 19323171

[11] Horne JB, van Herwerden L, Abellana S, McIlwain JL (2013) Observations of migrant exchange and mixing in a coral reef fish metapopulation link scales of marine population connectivity. J Hered 104: 532–546. 23580757 10.1093/jhered/est021

[12] Bell JJ (2008) The functional roles of marine sponges. Est Coast Shelf Sci 79: 341–353.

[13] Wulff JL (2001) Assessing and monitoring coral reef sponges: Why and how? Bull Mar Sci 69: 831–846.

[14] Webster NS, Taylor MW (2012) Marine sponges and their microbial symbionts: love and other relationships. Environ Micro 14: 335–346. 10.1111/j.1462-2920.2011.02460.x 21443739

[15] Knapp IS, Williams GJ, Carballo JL, Cruz-Barraza JA, Gardner JPA, et al (2012) Restriction of sponges to an atoll lagoon as a result of reduced environmental. Mar Poll Bull 66: 209–220. 10.1016/j.marpolbul.2012.08.017 23186728

[16] Bell JJ, Davy SK, Jones T, Taylor MW, Webster NS (2013a) Could some coral reefs become sponge reefs as our climate changes? Global Change Biology doi: 10.1111/gcb.12212. 10.1111/gcb.12212 23553821

[17] Powell A (2013) The impacts of predation and habitat degradation on coral reef sponges. Victoria University of Wellington PhD thesis.

[18] Duran S, Pascual M, Estoup A, Turon X (2004) Strong population structure in the marine sponge *Crambe crambe* (Poecilosclerida) as revealed by microsatellite markers. Mol Ecol 13: 511–522. 14871357 10.1046/j.1365-294x.2004.2080.x

[19] Whalan S, Johnson MS, Harvey E, Battershill C (2005) Mode of reproduction, recruitment, and genetic subdivision in the brooding sponge *Haliclona* sp. Mar Biol 146: 425–433.

[20] López-Legentil S, Pawlik JR (2009) Genetic structure of the Caribbean giant barrel sponge *Xestospongia muta* using the I3-M11 partition of COI. Coral Reefs 28: 157–165.

[21] Blanquer A, Uriz MJ, Caujapé-Castells J (2009) Small-scale spatial genetic structure in *Scopalina lophyropoda*, an encrusting sponge with philopatric larval dispersal and frequent fission and fusion events. Mar Ecol Prog Ser 380: 95–102.

[22] McMurray SE, Blum JE, Pawlik JR (2008) Redwood of the reef: growth and age of the giant barrel sponge, *Xestospongia muta* in the Florida Keys. Mar Biol 155: 159–171.

[23] McMurray SE, Henkel TP, Pawlik JR (2010) Demographics of increasing populations of the giant barrel sponge *Xestospongia muta* in the Florida Keys. Ecology 91: 560–570. 20392020 10.1890/08-2060.1

[24] López-Legentil S, Song BK, McMurray SE, Pawlik JR (2008) Bleaching and stress in coral reef ecosystems: hsp70 expression by the giant barrel sponge *Xestospongia muta* . Mol Ecol 17: 1840–1849. 18331247 10.1111/j.1365-294X.2008.03667.x

[25] Fromont J (1991) Descriptions of species of the Petrosida (Porifera: Demospongiae) occuring in the tropical waters of the Great Barrier Reef - The Beagle, Records of the Northern Territory of Museum of Arts and Science. 8(1): 73–96.

[26] Fromont J, Kerr S, Kerr R, Riddle M, Murphy P (1994) Chemotaxonomic relationships within, and comparisons between, the orders Haplosclerida and Petrosida (Porifera: Demospongiae) using sterol complements. Biochemic Syst Ecol 22(7): 735–752.

[27] Fromont J, Bergquist PR (1994) Reproductive biology of three sponge species of the genus *Xestospongia* (Porifera: Demospongiae: Petrosida) from the Great Barrier Reef. Coral Reefs 13(2): 119–126.

[28] Maldonado M (2006) The ecology of the sponge larva. Can J Zool 84: 175–94.

[29] Swierts T, Peijnenburg KT, de Leeuw C, Cleary DF, Hörnlein C, et al (2013) Lock, Stock and Two Different Barrels: Comparing the Genetic Composition of Morphotypes of the Indo-Pacific Sponge *Xestospongia testudinaria* . PloS one 8(9): e74396. 24069308 10.1371/journal.pone.0074396 PMC3771914

[30] Bell JJ, Smith DJ, Hannan D, Haris A, Thomas L (2013). Isolation and characterisation of twelve polymorphic microsatellite markers for *Xestospongia* spp. and their use for confirming species identity. Conservation Genetics Resources DOI 10.1007/s12686–013–0015–5.

[31] Clifton J, Unsworth RFK (2010) Introduction to the Wakatobi National Park. Chapter 1 In: Clifton J, Unsworth RKF, Smith DJ (eds) Marine Conservation and Research in the Coral Triangle: The Wakatobi National Park?. Nova Publishers, New York, p 1–9.

[32] Cullen LC (2010) Marine resource dependence and natural resource use patterns in a small Indo-Pacific island community: implications for management. Chapter 10 In: Clifton J, Unsworth RKF, Smith DJ (eds) Marine Research and Conservation in the Coral Triangle: the Wakatobi Marine National Park. Nova Publishers, New York, p 171–191.

[33] McMellor S, Smith DJ (2010) Coral reefs of the Wakatobi: abundance and biodiversity Chapter 2 In: Clifton J, Unsworth RKF, Smith DJ (eds) Marine research and conservation in the Coral Triangle: the Wakatobi National Park. Nova Science Publishers, New York, p 11–26.

[34] Holleley CE, Geerts PG (2009) Multiplex Manager 1.0: a cross-platform computer program that plans and optimizes multiplex PCR. BioTech 46: 511–517. 10.2144/000113156 19594450

[35] Nei M (1972) Genetic Distance between Populations. American Society of Naturalists 106: 283–292.

[36] Goudet J (1995) FSTAT (Version 1.2): A Computer Program to Calculate F-Statistics. Heredity 86: 485–486.

[37] Nei M (1987) Molecular Evolutionary Genetics (Columbia Univ. Press, New York). 512 p.

[38] Peakall R, Smouse PE (2006) GenAlEx 6: genetic analysis in Excel. Population genetic software for teaching and research. Mol Ecol Notes 6: 288–295. 10.1093/bioinformatics/bts460 PMC3463245 22820204

[39] Excoffier L, Laval G, Schneider S (2005) Arlequin ver. 3.0: An integrated software package for population genetics data analysis. Evolutionary Biology Online 1: 47–50. PMC2658868 19325852

[40] Rice WR (1989) Analyzing tables of statistical tests. Evolution 43: 223–235. 28568501 10.1111/j.1558-5646.1989.tb04220.x

[41] Weir BS, Cockerham CC (1984) Estimating F-statistics for the analysis of population structure. Evolution 1358–1370. 10.1111/j.1558-5646.1984.tb05657.x 28563791

[42] Meirmans PG, Hedrick PW (2011) Assessing population structure: F(ST) and related measures. Mol Ecol Res 11: 5–18. 10.1111/j.1755-0998.2010.02927.x 21429096

[43] Pritchard JK, Stephens M, Donnelly P (2000) Inference of population structure using multilocus genotype data. Genetics 155: 945–959. 10835412 10.1093/genetics/155.2.945 PMC1461096

[44] Evanno G, Regnaut S, Goudet J (2005) Detecting the number of clusters of individuals using the software STRUCTURE: a simulation study. Mol Ecol 14: 2611–2620. 15969739 10.1111/j.1365-294X.2005.02553.x

[45] Earl DA (2012) STRUCTURE HARVESTER: a website and program for visualizing STRUCTURE output and implementing the Evanno method. Cons Gen Res 4: 359–361.

[46] Jakobsson M, Rosenburg NA (2007) CLUMPP: a cluster matching and permutation program for dealing with label switching and multimodality in analysis of population structure. Bioinformatics 23: 1801–1806. 17485429 10.1093/bioinformatics/btm233

[47] Rosenberg NA (2004) DISTRUCT: a program for the graphical display of population structure. Mol Ecol Notes 4: 137–138.

[48] Rannala B, Mountain JL (1997) Detecting immigration by using multilocus genotypes. Proc Nat Acad Sci 94: 9197–9201. 9256459 10.1073/pnas.94.17.9197 PMC23111

[49] Piry S, Alapetite A, Cornuet JM, Paetkau D, Baudouin L, et al (2004) GeneClass2: A Software for Genetic Assignment and First-Generation Migrant Detection. Heredity 95: 536–539. 10.1093/jhered/esh074 15475402

[50] Berry O, Tocher MD, Sarre SD (2004) Can assignment tests measure dispersal? Mol Ecol 13: 551–561. 14871360 10.1046/j.1365-294x.2004.2081.x

[51] Paetkau D, Slade R, Burden M, Estoup A (2004) Direct, real-time estimation of migration rate using assignment methods: a simulation-based exploration of accuracy and power. Mol Ecol 13: 55–65. 14653788 10.1046/j.1365-294x.2004.02008.x

[52] Peakall R, Ruibal M, Lindenmayer DB (2003) Spatial autocorrelation analysis offers new insights into gene flow in the Australian bush rat, *Rattus fuscipes* . Evolution 57: 1182–1195. 12836834 10.1111/j.0014-3820.2003.tb00327.x

[53] Miller KJ, Ayre DJ (2008) Population structure is not a simple function of reproductive mode and larval type: insights from tropical corals. J Anim Ecol 77(4): 713–724. 18422556 10.1111/j.1365-2656.2008.01387.x

[54] Wright S (1931) Evolution in Mendelian populations. Genetics 16: 97–159. 17246615 10.1093/genetics/16.2.97 PMC1201091

[55] Wright S (1943) Isolation by Distance. Genetics 28: 114–38. 17247074 10.1093/genetics/28.2.114 PMC1209196

[56] Almany GR, Berumen ML, Thorrold SR, Planes S, Jones GP (2007) Local replenishment of coral reef fish populations in a marine reserve. Science 316: 742–744. 17478720 10.1126/science.1140597

[57] Selkoe KA, Toonen RJ (2006) Microsatellites for ecologists: a practical guide to using and evaluating microsatellite markers. Ecol Letters 9: 615–629. 10.1111/j.1461-0248.2006.00889.x 16643306

[58] Bell JJ, Smith DJ (2004) Ecology of sponge assemblages (Porifera) in the Wakatobi region, south-east Sulawesi, Indonesia: Richness and abundance. J Mar Biol Assoc UK 84: 581–591.

[59] Bell JJ (2007) Contrasting patterns of species and functional composition of coral reef sponge assemblages. Mar Ecol Prog Ser 339: 73–81.

[60] Kinlan BP, Gaines SD (2003) Propagule dispersal in marine and terrestrial environments: a community perspective. Ecology 84: 2007–2020.

